# Brain structure is linked to the association between family environment and behavioral problems in children in the ABCD study

**DOI:** 10.1038/s41467-021-23994-0

**Published:** 2021-06-18

**Authors:** Weikang Gong, Edmund T. Rolls, Jingnan Du, Jianfeng Feng, Wei Cheng

**Affiliations:** 1 Centre for Functional MRI of the Brain (FMRIB), Nuffield Department of Clinical Neurosciences, Wellcome Centre for Integrative Neuroimaging, University of Oxford, Oxford, UK; 2 Institute of Science and Technology for Brain-inspired Intelligence, Fudan University, Shanghai, China; 3 Key Laboratory of Computational Neuroscience and Brain-Inspired Intelligence, Fudan University, Ministry of Education, Shanghai, China; 4 Department of Computer Science, University of Warwick, Coventry, UK; 5 Oxford Centre for Computational Neuroscience, Oxford, UK; 6 Fudan ISTBI—ZJNU Algorithm Centre for Brain-inspired Intelligence, Zhejiang Normal University, Jinhua, China; 7 MOE Frontiers Center for Brain Science, Fudan University, Shanghai, China; 8 Zhangjiang Fudan International Innovation Center, Shanghai, China

**Keywords:** Neuroscience, Cognitive neuroscience

## Abstract

Children’s behavioral problems have been associated with their family environments. Here, we investigate whether specific features of brain structures could relate to this link. Using structural magnetic resonance imaging of 8756 children aged 9-11 from the Adolescent Brain Cognitive Developmental study, we show that high family conflict and low parental monitoring scores are associated with children’s behavioral problems, as well as with smaller cortical areas of the orbitofrontal cortex, anterior cingulate cortex, and middle temporal gyrus. A longitudinal analysis indicates that psychiatric problems scores are associated with increased family conflict and decreased parental monitoring 1 year later, and mediate associations between the reduced cortical areas and family conflict, and parental monitoring scores. These results emphasize the relationships between the brain structure of children, their family environments, and their behavioral problems.

## Abstract (web summary)

Child behavior has been associated with parenting behavior. Here, the authors investigate associations between child behavior, parental behavior, and structural MRI using the Adolescent Brain Cognitive Developmental (ABCD) study dataset.

## Introduction

Parental behavior and parent–child relationships play an important role in childrens’ physical, cognitive, and mental development, as well as predicting patterns of future adolescent brain development^1^. Population-based studies have shown that parental behaviors are associated with the prevalence of social, emotional, behavioral problems^2–4^, and mental illness in the children^5–7^. Consistent with this, conflict in the family is associated with emotional, behavioral, and social outcome and depression in the children^8,9^. Parental behaviors and conflict are also associated with the childrens’ cognitive control and risk-taking behaviors^10^, cognitive skills and academic performance^8^, and pubertal timing^11^. Although in many of these studies it is assumed that the parental behavior influences the children’s conduct problems, it has also been reported that adolescent conduct problems predicted changes over time in the parental monitoring behaviors, suggesting that parental behavior can also potentially be a reaction to and not only a predictor of conduct problems in adolescence^7^.

A substantial body of literature has also documented the link between parental behaviors and brain structure and function in children^12–15^. One longitudinal study reported that positive maternal parenting can reduce the impact of the neighborhood and socioeconomic disadvantage on the development of brain structure in adolescents^13^. Another study showed that supportive parenting can predict larger hippocampal volume in children^16^. In addition, parental behaviors also have an impact on brain development. For example, adults with a childhood trauma history, such as exposure to major family disturbances, have reduced gray matter volume in prefrontal-limbic brain regions^17^, and an altered structural connectome across many brain regions including the temporal pole, the insula, and the ventromedial prefrontal cortex^18,19^. However, most of the previous investigations were only based on small-sized and cross-sectional datasets. In addition, none of the previous neuroimaging studies have compared the association of scores of parental monitoring and family conflict with brain structure in the children. Moreover, how neural differences in the children and their mental health and cognition are related to the family environment have still not been explored comprehensively.

The literature cited above shows that high parental monitoring is associated with reduced social, emotional, and behavioral problems in the children. Parental monitoring was measured in some previous studies with a parent–child interaction task^13,16^; in the present study, parental monitoring was measured by scores on questions such as “How often do your parents/guardians know where you are?” as shown in the Supplementary Material^20,21^. Conflict in the family and between parents is associated with emotional, behavioral, and social outcomes and depression in children, and with cognitive skills, cognitive control, and academic performance in children. It is not yet fully established whether some of these associations reflect family effects on the children or childrens’ effects on the families. There is also evidence that differences in the brains of the children are related to these associations between the family environment and the children’s behavior^12–19^.

**Fig. 1:**
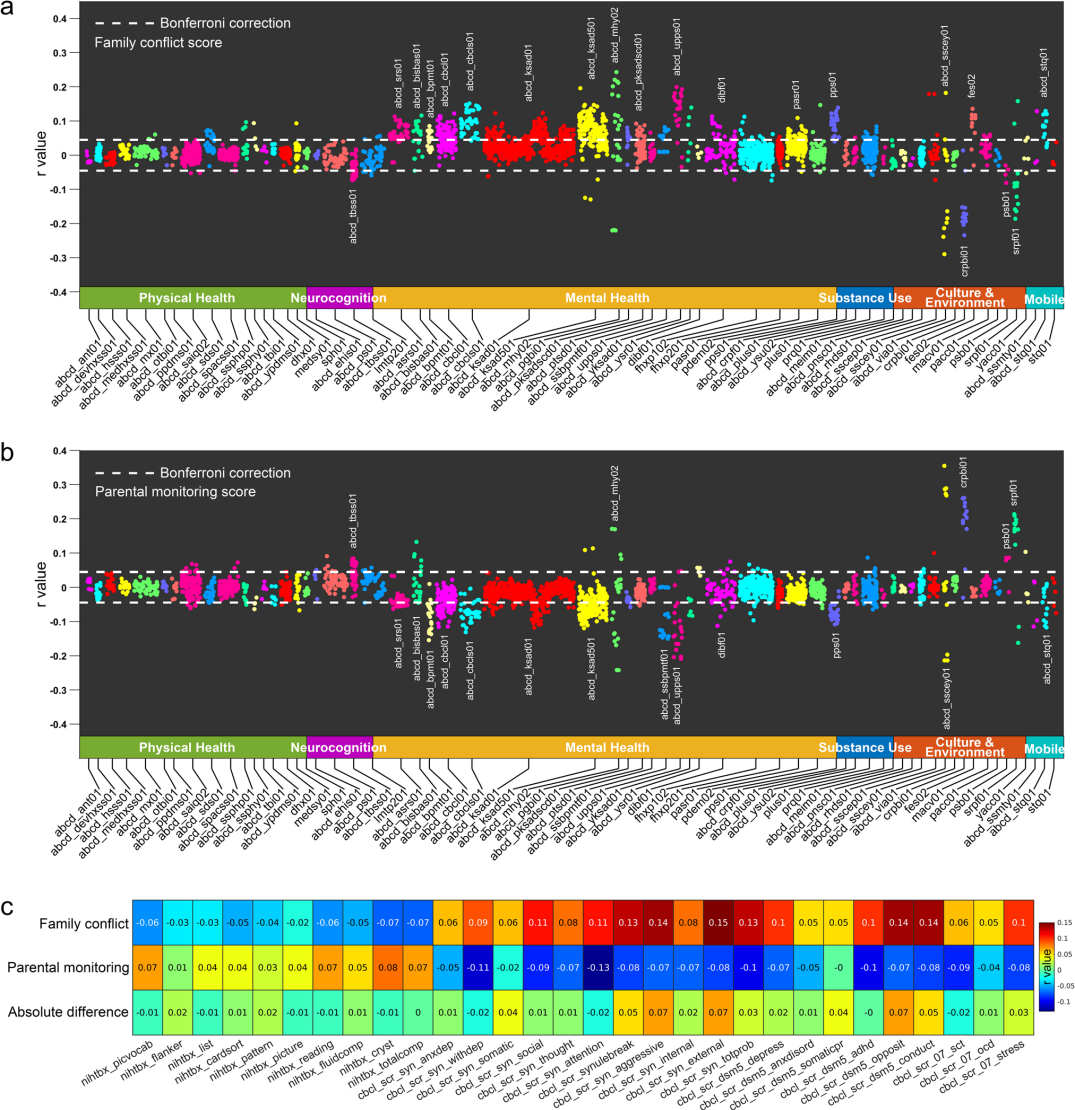
Behavior-level correlation analysis. **a** Correlation between the family conflict score and behavioral measures in the ABCD. The behavioral measures most associated with family conflict relate to mental health. The behavioral measures and corresponding abbreviations are defined in the “Methods” section and Table S1. **b** Correlation between the parental monitoring score and behavioral measures in the ABCD. **c** Correlation of the family conflict (row 1) and the parental monitoring score (row 2) with the cognitive and behavioral scores. Row 3 shows the absolute difference of the correlation between rows 1 and 2. All measures were significantly correlated with the family conflict score except nihtbx_flanker, nihtbx_list, and nihtbx_picture and all measures were significantly correlated with the parental monitoring score except nihtbx_flanker, nihtbx_pattern, cbcl_scr_syn_somatic, and cbcl_scr_dsm5_somaticpr (Bonferroni correction, *p* < 0.05).

Here, we performed a large-scale investigation of the relationships between family environment (based on self-reported scales of family conflict and parental monitoring that are available within the Adolescent Brain Cognitive Developmental (ABCD) dataset and are shown in the Supplementary Material) and childrens’ behavioral problems and cognitive scores, and brain structure, using data from 8756 children from the ABCD study. In this study, we set out to answer the following questions: (1) What is the relationship between child reported family conflict scores and parental monitoring scores, and parent-reported behavioral problems as assessed by the Child Behavior Checklist (CBCL) in the children? Family conflict and parental monitoring scores were used because these summarize many subscores (of questions related to the family environment) used in the ABCD dataset, and are established measures for assessing the family environment^20,21^. Behavioral problems and cognitive scores were also used because they summarize measures from established scales. (2) What are the relations between the family conflict and parental monitoring scores, and the cognitive scores in the children? (3) Using a longitudinal design, are differences after 1 year for the family conflict and parental monitoring scores related to the children’s behavioral problem scores 1 year earlier or are associations found in both directions? Both directions were tested, as there is some evidence for effects in different directions in the literature^7^. (4) What is the relationship between scores of family conflict and parental monitoring and brain structure in the children? A voxel-level analysis was performed to assess this, and results are presented in the main text for the area of different cortical regions. The area and volume of cortical regions is strongly correlated in the ABCD dataset^22^, and we chose to present the results for area in the main text and volume in the Supplementary Material. (5) In the light of the longitudinal association analyses, we tested the hypothesis that behavioral problems in children mediate in part the associations between the reduced cortical areas and the increased family conflict scores, and that behavioral problems in children mediate in part the associations between the increased cortical areas and the higher parental monitoring scores. (6) Because depressive symptoms are sometimes associated with the family environment^9,16^ and with changes in the brain^23–28^, measures of depression were included in this investigation (as measured by diagnoses using Kiddie Schedule for Affective Disorders and Schizophrenia (KSADS) shown in Table S2 or depressive symptoms measured by the CBCL shown in Fig. 1). We measured which of the 20 behavioral problems scores and 10 cognitive scores were most associated with the family conflict and parental monitoring scores, to help in future assessments of children.

Here, we show that structural differences, for example, in the orbitofrontal and cingulate cortices of the children, are linked to the associations between family behavior scores and scores of behavioral problems in the children.

## Results

### Association of family conflict and parental monitoring scores with other behavioral scores

We first correlated family conflict and parental monitoring scores with a broad range of behavioral measures (listed in Table S1) in the children of the sample, with potential confounds regressed out (see “Methods” section). The CBCL measures for behavioral problems in the children (abcd_cbcl01) were significantly positively correlated with family conflict score (Fig. 1a). Overall, 45 out of 120 items were significant (Bonferroni correction, *p* < 0.05). Overall, 17 out of the 49 neurocognition measures (abcd_tbss01) were negatively correlated with higher family conflict scores (Bonferroni corrected, *p* < 0.05, Fig. 1a). Overall, 31 out of the 45 lifetime mental disorder diagnoses scores based on the KSADS assessment (abcd_ksad501)^29^ were significantly associated with the family conflict scores (Table S2). Screen time utilization (e.g., mobile phone, TV, internet, and video games) (abcd_stq01), behavioral inhibition and activation (abcd_bisbas01), mental health (abcd_ksad01, abcd_ksad501, abcd_mhy02), conduct disorder (abcd_pksadscd01), impulsivity (abcd_upps01), school, family, social relations (dibf01), and prodromal psychosis levels (pps01) were all positively correlated with family conflict score (Fig. 1a). The school risk and protective factors (srpf01) were negatively correlated with family conflict score (Fig. 1a). Similar behavioral measures in the children were significantly negatively correlated with the parental monitoring scores, that is, low CBCL measures in the children were correlated with high parental monitoring scores (Fig. 1b).

We then estimated the “raw” correlations between the two family environment scores (family conflict and parental monitoring) based on youth surveys, and calculated the correlations with other important parent-related variables based on parent surveys (see “Methods” section). The correlation between the family conflict scores and parental monitoring scores was −0.23 (*p* = 4.5 × 10^−107^). The correlation of the family conflict scores with the parents’ income was −0.14 (*p* = 3.8 × 10^−37^), with the parents’ years of education was −0.11 (*p* = 5.7 × 10^−25^), and with the fathers’ and mothers’ psychiatric history were 0.013 (*p* = 0.23) and 0.023 (*p* = 0.03). The correlations of the parental monitoring score with the parents’ income was 0.12 (*p* = 6.5 × 10^−28^), with the parents’ years of education was 0.10 (*p* = 1.3 × 10^−19^), and with the fathers’ and mothers’ psychiatric history were −0.007 (*p* = 0.51) and −0.028 (*p* = 0.01).

### Association between family conflict and parental monitoring scores with child cognitive and behavioral scores

Next, we correlated both family conflict and parental monitoring scores with cognitive scores, and behavioral problems measured with the CBCL, with potential confounds regressed out (see “Methods” section).

The family conflict scores were negatively correlated with all the cognitive scores (Fig. 1c). The range of correlations between the family conflict scores and the cognitive measurements was between −0.02 and −0.08 (Fig. 1c). Thus, children in families with a high family conflict score had poorer cognitive performance. Further, the family conflict scores were positively correlated with all the individual behavioral problems scores in the children with *r* values ranging from 0.05 to 0.15 (Fig. 1c). Scores for aggressive (*r* = 0.15), external (*r* = 0.15), conduct (*r* = 0.14), and opposition behaviors (*r* = 0.14) were most significantly correlated with the family conflict scores (Bonferroni corrected, *p* < 0.05). Children in families with high family conflict scores tended to have higher total behavioral problems scores (*r* = 0.13, Bonferroni corrected *p* < 0.05).

The parental monitoring scores were positively correlated with all of the cognitive scores (Fig. 1c). The range of correlations between the parental monitoring scores and cognitive measurements was between 0.03 and 0.09 (Fig. 1c). Thus, children in families with a high parental monitoring score have high cognitive scores. Further correlation analysis also showed that all the behavioral problem scores were negatively correlated with the parental monitoring scores with *r* values ranging from −0.01 to −0.13 (Fig. 1c). The attention (*r* = −0.13) and depressive (*r* = −0.11) problems scores were those most significantly negatively correlated with the parental monitoring scores (Bonferroni corrected, *p* < 0.05). The children in families with high parental monitoring scores had significantly lower total behavioral problems scores (*r* = −0.10, Bonferroni corrected *p* < 0.05).

### Family conflict score is associated with lower cortical area, and parental monitoring score is associated with higher cortical area in children

We performed vertex-wise association analysis of cortical areas in the children with both the family conflict scores and parental monitoring scores, with potential confounds regressed out (see “Methods” section).

**Fig. 2:**
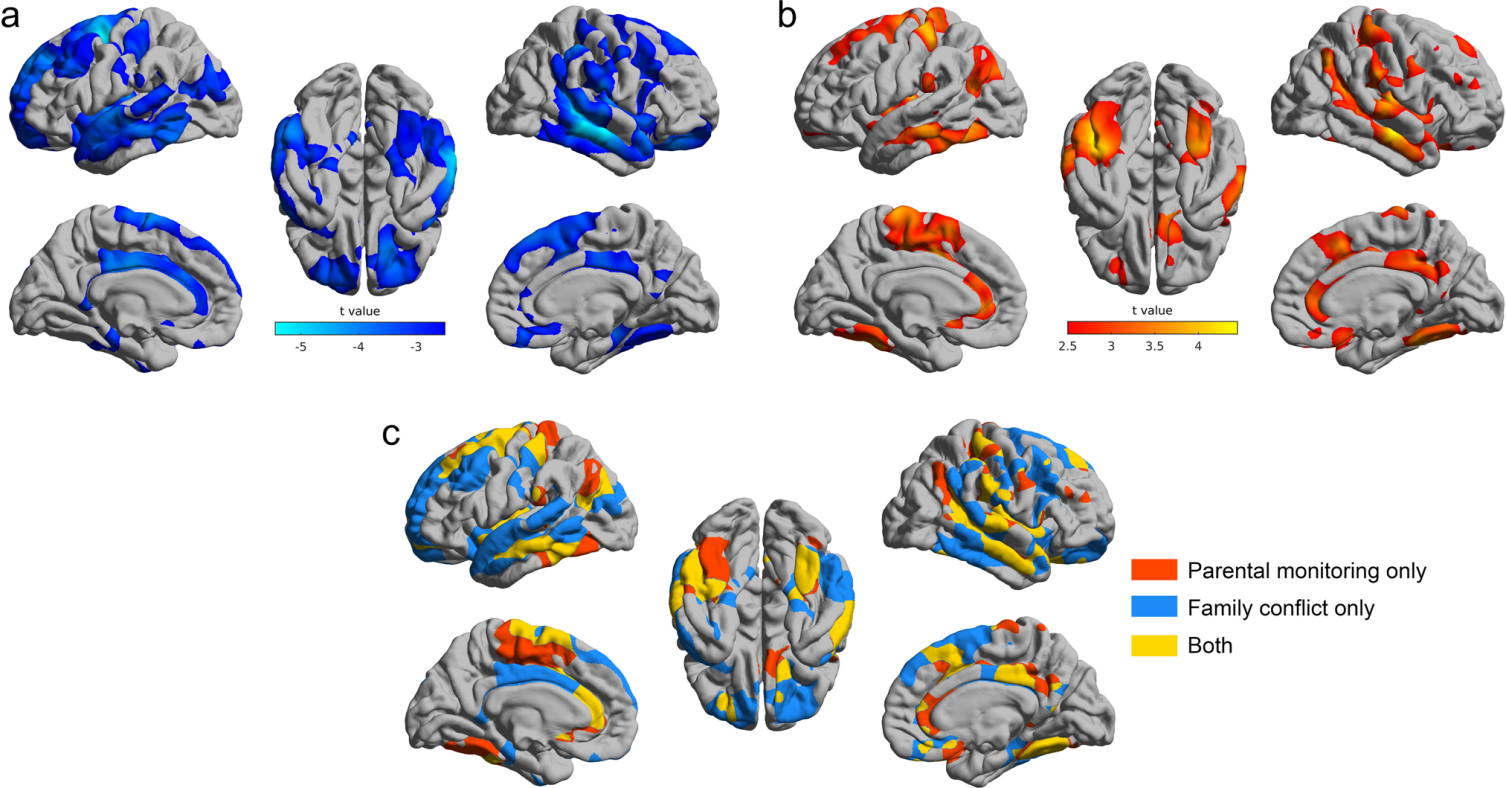
The brain regions with their cortical areas correlated with the family conflict score and parental monitoring score. **a** The areas of brain regions that were significantly correlated with the family conflict score (FDR *p* < 0.05 corrected). The blue color indicates that lower surface areas were correlated with more severe family conflict scores. **b** The areas of brain regions that were significantly correlated with the parental monitoring score (FDR *p* < 0.05 corrected). The red color indicates higher surface areas were correlated with more positive parental monitoring. **c** The brain regions significantly (FDR p < 0.05 corrected) related to parental monitoring only (red color), family conflict only (blue color), and the brain regions correlated with both measurements (yellow color).

The total cortical area was significantly negatively correlated with the family conflict scores (*r* = −0.048, *p* = 6.2 × 10^−6^). Specifically, in children with high family conflict scores, vertex-wise analysis showed that the cortical areas were lower of the orbitofrontal cortex, middle temporal gyrus, supracallosal anterior cingulate cortex, and superior prefrontal cortex (FDR corrected, *p* < 0.05, Fig. 2a).

Total cortical area was significantly positively correlated with the parental monitoring scores (*r* = 0.038, *p* = 4.3 × 10^−4^). Specifically, in children with high parental monitoring scores, vertex-wise analysis showed that the cortical areas were higher of the anterior and posterior cingulate cortex, middle temporal gyrus, angular/supramarginal gyrus, and supplementary motor areas (FDR corrected, *p* < 0.05, Fig. 2b).

The brain areas correlated with both family conflict and parental monitoring scores are shown in Fig. 2c in yellow, and included the anterior and posterior cingulate cortex, the middle and inferior temporal gyri, and some postcentral and related areas.

In Fig. S1, we show that these associations are robust with respect to random choice of the siblings (see “Methods” section). In Fig. S2, we show the corresponding results for cortical volume, which are consistent with those for cortical area, though the significant regions are less extensive for volume. In Fig. S3, we show that the associations of cortical area and cortical volume with the family environment scores are high (*r* = 0.75, *p* < 10^−10^ for both scores) (Fig. S3A, B), and cortical area and volume themselves also have a high correlation (*r* = 0.84, *p* < 10^−10^), as also found previously^30^ (Fig. S3C). Cortical thickness was not associated with the family conflict and parental monitoring scores (all FDR corrected *p* > 0.05), and for brevity is not described further.

### Longitudinal association between the childrens’ behavioral problems scores, and the family conflict and parental monitoring scores

**Fig. 3:**
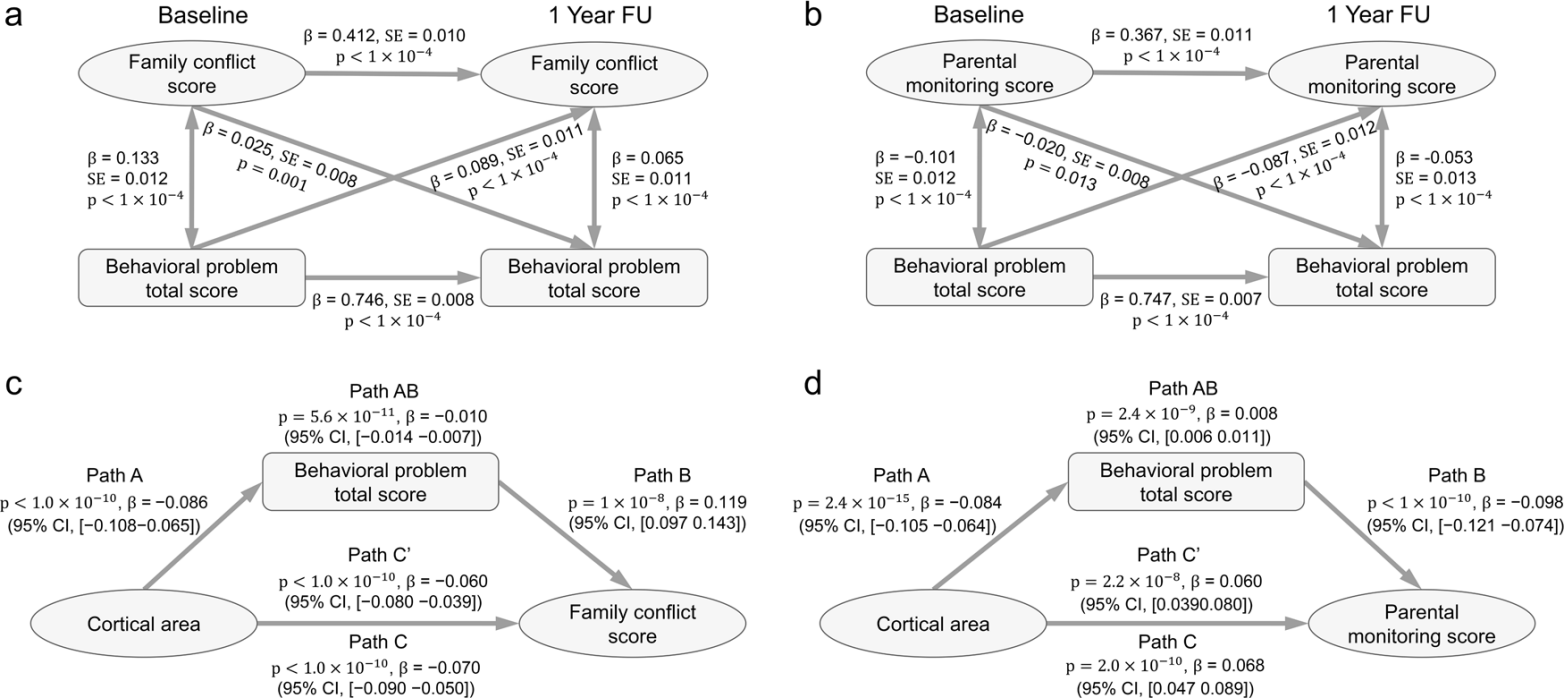
The longitudinal association and mediation analyses. **a** The longitudinal association between the behavioral problems total score (TotProb CBCL Syndrome Scale) in the children and the family conflict score measured 1 year later using structural equation modeling. Higher behavioral total scores were associated with higher family conflict scores 1 year later (*p* < 1.0 × 10^−4^, *n* = 8836), and higher family conflict scores were associated with higher behavioral problems  total scores 1 year later (*p* = 0.001, *n* = 8836), but less significantly. **b** The longitudinal association between the behavioral problems  total score in the children and the parental monitoring score using structural equation modeling. Higher behavioral problems  total scores were associated with lower parental monitoring scores 1 year later (*p* < 1.0 × 10^−4^, *n* = 8836) (solid diagonal line), and higher parental monitoring scores were associated with lower behavioral problems  total scores 1 year later (*p* = 0.013, *n* = 8836), but less significantly. **c** Mediation analysis: the indirect path (A, AB, and B) shows that the behavioral problems total score in the children significantly mediates the association between the cortical area in the children and the family conflict scores (*β* = −0.01, *p* = 5.6 × 10^−11^, *n* = 8756, 14.7% variance explained). Path A: Association between the independent variable (the cortical area) and the mediator (the behavioral problems total score). Path B: association between the mediator (the behavioral problems total score) and the outcome (the family conflict score). Path C’ shows a significant reduction in the regression coefficient between the cortical area and the family conflict score when the mediator (the behavioral problems total score) is taken into account, with the association without this mediation shown in path C. Path AB indicates the extent to which taking the behavioral problems total score into account can explain 14.7% of the total variances between the cortical area in the children and the family conflict score, which is significant as noted above at *p* = 5.6 × 10 ^−11^. (The variance explained is obtained by dividing β in path AB by β in path C.) **d** A corresponding mediation analysis showed that the behavioral problems total score in the children significantly mediates the association between the cortical area in the children and the parental monitoring score (*β* = 0.008, *p* = 2.4 × 10^−9^, *n* = 8756, 12.1% variance explained). All statistical tests here are two-sided, and pass Bonferroni correction (*p* < 0.05).

We performed longitudinal association analyses between the children’s behavioral problems (CBCL) scores and the family conflict scores/parental monitoring scores. Structural equation modeling was used to analyze the changes of scores between the baseline ages and 1 year later, with potential confounds regressed out (see “Methods” section). The childrens’ total behavioral problems scores at the baseline age were significantly associated with increased family conflict ($\beta =0.089$, *p* < 10^−4^) and decreased parental monitoring ($\beta =-0.087$, *p* < 10^−4^) at the 1-year follow-up (Fig. 3a, b). The reverse was less significant for family conflict ($\beta =0.025$, *p* = 0.001) and parental monitoring ($\beta =-0.020$, *p* = 0.013). The permutation test showed that the effect of the association between the children’s behavioral problems scores at the baseline age, and family conflict and parental monitoring at the 1-year follow-up, was significantly higher than the reverse effect (*p* < 0.001). The model accounted for 24% of the variance in the family conflict scores, and 40.4% of the variance in the parental monitoring scores at the 1-year follow-up, by taking into account the childrens’ total behavioral problems scores at baseline (i.e., 1 year earlier). Most of the individual behavioral problems subscores had significant longitudinal associations as shown in Tables S3 and S4.

### Mediation analysis

In the light of the longitudinal association analyses, which showed a bidirectional effect of child behavioral problems on family conflict, we performed a mediation analysis to test a first hypothesis that the behavioral problems mediated the association between the reduced cortical area and the increased family conflict score. It was found that the children’s behavioral problems total score (CBCL) did significantly mediate the relationship between the cortical areas (shown in Fig. 2a) and the family conflict scores (Fig. 3a, path AB, 14.7% of the total effect size, *β* = −0.01; *p* = 5.6 × 10^−11^; 95% CI, −0.014 to −0.007). The first hypothesis was thus supported by this analysis. Most of the behavioral problems subscores had significant mediation effects as shown in Table S5.

The second hypothesis was that the children’s behavioral problems scores  mediate in part the associations between the increased cortical areas and the higher parental monitoring scores. It was found that the childrens’ behavioral problems total score did significantly mediate the relationship between the cortical areas (shown in Fig. 2b) and the parental monitoring scores (Fig. 3b, path AB, 12.1% of the total effect size, *β* = 0.008; *p* = 2.4 × 10^−9^; 95% CI, 0.006–0.011). The second hypothesis was thus also supported by this analysis. Most of the behavioral problems subscores had significant mediation effects as shown in Table S6. Because the longitudinal analysis showed an effect in both directions between child behavioral problems and parental monitoring, we also tested a mediation model for whether the cortical areas mediate the association between the parental monitoring scores and behavioral problem scores. We found a significant effect as shown in Fig. S4.

Both of these mediation analyses were performed with data at the baseline time. Potential confounds were regressed out (see “Methods” section). We also tested whether the mediation effect remained if the change of the parents’ psychiatric symptom total score (ABCD Parent Adult Self Report Scores Aseba, abcd_asrs01) was included as an additional covariate. The results in Fig. S5 show that the mediation models were still significant.

## Discussion

This research demonstrates in a large sample the association between the area of certain cortical regions and scores related to family environment, including family conflict and parental monitoring, and shows that the childrens’ behavioral problems (assessed with the CBCL) mediate the associations between the structure of brain regions and the family problems. Moreover the behavioral problems in the children relate in part to the parental monitoring and the family conflict that are measured 1 year later. The associations involving the area of cortical regions such as the anterior cingulate and orbitofrontal cortex provide evidence about brain regions that are involved in the associations between the childrens’ behavioral problems and the family conflict and parental monitoring. The findings also raise the importance of measuring and assessing the effects of the childrens’ behavioral problems on the family, as well as the more traditional concepts of how the parents influence the children.

For clarity, we assess here what the results show about the family conflict scores. The behavioral problems scores of the 8756 children aged 9–11 years were positively correlated with the family conflict scores. The family conflict scores were also correlated with smaller areas of some cortical regions, including the orbitofrontal cortex, middle temporal gyrus, supracallosal anterior cingulate cortex, and superior prefrontal cortex (Fig. 2a). The longitudinal analysis showed that the childrens’ total behavioral problems scores at the baseline age were significantly associated with increased family conflict at the 1-year follow-up. The reverse was less significant. The childrens’ behavioral problems significantly mediated the association between the lower areas of cortical regions and the increased family conflict scores. The cognitive scores of the children were negatively correlated with the family conflict scores and positively correlated with the parental monitoring scores. Our interpretation of these findings is that behavioral problems mediate effects of reduced cortical areas of some key brain regions including the orbitofrontal and anterior cingulate cortices in the 9-11 years  old children on the higher family conflict scores. There is evidence that these brain areas are associated with behavioral problems such as depression^31–34^. However, we cannot exclude mediation effects in the other direction, such as an effect of the higher family conflict scores on the lower cortical areas. We also note that the family conflict scores used here (see Supplementary Information) take into account the whole family and not specifically parents, and so it is not unexpected that these family based scores are associated with child behaviour. What we believe is interesting is that in this study we show that cortical area is associated with these processes.

Second, we consider what the results show about the parental monitoring scores, where high parental monitoring includes for example discussion and social interaction with the children (see Supplementary Material). Lower behavioral problems scores in the children were positively correlated with the parental monitoring scores. The longitudinal analysis showed that childrens’ high total behavioral problems scores at the baseline age were significantly associated with decreased parental monitoring scores at the 1-year follow-up. The reverse was less significant. The childrens’ behavioral problems significantly mediated the association between the lower areas of cortical regions (including the anterior and posterior cingulate cortex, middle temporal gyrus, angular/supramarginal gyrus, and supplementary motor areas) and the decreased parental monitoring scores. In addition, the cortical areas mediate the association between the parental monitoring scores and behavioral problems scores. The cognitive scores of the children were positively correlated with the parental monitoring scores. Again, it is especially interesting that cortical areas in the children are involved in these processes that are related to parental monitoring. Further, although frequently the effects of parental influences on the children are considered^35^, influences of the childrens’ behavior on the parents have been described^7^, with the present study extending this by showing how some brain regions in the children and some behavioral problems are linked to the influences of the children on parental monitoring behavior.

Third, we compared two family environment scores. The family conflict and parental monitoring scores were correlated only −0.23 across the 8756 participants. This implies that they are not simple opposites, but that these two measures reflect something at least partly different. The family conflict scores were more associated with the aggressive, external, conduct, and opposition scores in the children. The parental monitoring scores were positively correlated with low attention problems and low depressive problems scores. High areas of the pregenual anterior cingulate cortex are associated with high parental monitoring (Fig. 2b). This is the part of the cingulate cortex associated with rewards, and has correlations with the pleasantness of stimuli^36–39^. Low areas of the supracallosal anterior cingulate cortex (middle anterior and middle posterior part of the cingulate gyrus and sulcus) are associated with high family conflict (Fig. 2a). This is the part of the cingulate cortex associated with punishment and nonreward, and has correlations with the unpleasantness of stimuli^36,37^. We thus propose that these differences between the parental monitoring and the family conflict relate to the different functions of these different parts of the anterior cingulate cortex. Correspondingly, the medial orbitofrontal cortex (involved in reward^40–42^) had a low area (Fig. 2a) that was associated with family conflict which in turn was associated with behaviors that included aggressive and external behaviors.

We found that cortical thickness was not associated with the family conflict and parental monitoring scores. Consistent with this, it has been reported that cortical area rather than thickness in children is associated with sleep duration and associated cognitive and behavioral problems^22^, and with lead-exposure risk^43^. A possible explanation is that the overall regional pattern of cortical thickness is relatively stable from early postnatal life, while the changes of cortical area have more development and changes later^30^. Other possible explanations are that cortical surface area and cortical thickness have distinct sources of genetic influence^44^, and that cortical thickness is more polygenic than cortical area^45^.

The longitudinal analysis showed that the childrens’ behavioral problems score was associated with parental monitoring 1 year later (*p* < 1 × 10^−4^). The reverse association was also found, but it was less significant (*p* = 0.013). The mediation analysis provided evidence that the childrens’ behavioral problems scores mediate the association between increased cortical area in the children and the parental monitoring. Our large-scale longitudinal analysis with 8836  children suggests that high behavioral problems of the children are associated with the family conflict, and low behavioral problems in the children are associated with high parental monitoring. A precedent for thinking that there are at least some effects from the childrens’ behavior to the parents is that a “bidirectional” model may be appropriate, i.e., children influence parents and parents influence children^46,47^. For example, it has been suggested that children may deliberately intervene to change parental behaviors, beliefs, and attitudes^47^. We note that the longitudinal cross-lagged panel model (CLPM) accounts for temporal stability through the inclusion of autoregressive parameters and cannot separate effects within and between individuals, so our findings indicate the group average effect, not effects at the individual level^48^.

To further understand the associations described here between the childrens’ psychiatric problems and the family conflict and parental monitoring, we analyzed whether there are associations between the relatives’ psychiatric history and the behavioral problems in the children. We found that the relatives’ psychiatric history was significantly positively correlated (*r* = 0.14, *p* = 3 × 10^−47^) with the childrens’ total psychiatric problems score. This is consistent with the evidence that there is a genetic component to psychiatric problems in children^49,50^. A possible explanation for our findings is that there is a genetic contribution to brain structure in children that can affect their behavior and cognition, and that these behavioral/psychiatric and cognitive states can influence the family problems and parental monitoring measure of the family environment. However, we stress that the role of gene–environment interactions is complex^51^, and our study reports associations, and so is not able to assess causality. We also stress that associations between child behavioral problems and parenting or family conflict constructs could reflect a number of possibilities. For example, this could reflect either passive gene–environment interaction, where parents and children share genetic predispositions, or evocative gene–environment interaction, where children’s behavioral problems elicit family conflict. We do emphasize that in all the analyses described here, the parents’ psychiatric history was regressed out, so that possible contribution of genetic effects has been taken into account in the analyses.

Despite these limitations we believe our study has a number of strengths. It includes voxel-level analysis, which allows the exact brain regions to be delineated, rather than large predefined regions as in many investigations of relations involving brain areas. This sample is large, and the ABCD dataset includes many behavioral measures. The children in this dataset are of almost the same age thereby controlling for age. The longitudinal design of the study has enabled us to analyze the relationship between psychiatric problems in the children, and the family environment measured 1 year later. Overall, we have found an association between childrens’ behavior and cognition, and parental monitoring and family conflict that is partly mediated by differences in the area and volume of brain regions that include the orbitofrontal cortex and anterior cingulate cortex, brain regions that are involved in emotion and its links to action^36,38,42,52^.

## Methods

### Participants and data preprocessing

The neuroimaging and behavioral data used in this paper were obtained from the ABCD Dataset Data Release 3.0 (https://abcdstudy.org/scientists/data-sharing/). A total of 11,875 participants aged between 9 and 11 years were obtained from the ABCD study, which was a large longitudinal study that recruited children across 21 research sites across the USA^53^. The ABCD investigators obtained written and oral informed consent from parents and children, respectively^54^. More details of the subjects, and the collection and preprocessing parameters of the data are provided at the ABCD website (https://abcdstudy.org/scientists/protocols/) and elsewhere^53,55^.

The minimal preprocessed T1- and T2-weighted structural images were downloaded from the ABCD study. The minimal preprocessing done by the ABCD team includes: Raw T1-weighted and T2-weighted structural images were corrected for gradient nonlinearity distortions; T2-weighted images were registered to T1-weighted images using mutual information after coarse, rigid-body pre alignment via within-modality registration to atlas brains; intensity inhomogeneity correction was performed by applying smoothly varying, estimated B1-bias fields; and images were rigidly registered and resampled into alignment with an averaged reference brain in standard space. We used FreeSurfer v6.0 to preprocess the structural MRI images, including cortical surface reconstruction, subcortical segmentation, and estimation of morphometric measures, i.e., cortical area, thickness, and volume, using both T1- and T2-weighted MRI images. To perform vertex-level group analysis, the cortical surface of each subject was registered to a standard fsaverage space and the morphometric measures were smoothed by a Gaussian kernel (FWHM = 10 mm). All preprocessing followed the pipeline used by the ABCD^55^. The differences were that we have used a newer version of FreeSurfer and both T1- and T2-weighted images for surface-based analysis^55^. A total of 10,883 images were successfully preprocessed by FreeSurfer. Quality control was then performed using software Qoala-T^56^. Overall, 121 MRI images with scores below 40 were removed from the study, as recommended^56^. As there were correlated observations within families due to twins and siblings and at sites, we picked only one child in each family to eliminate this correlation effect in the subsequent statistical modeling, resulting in 9117 children. (The results are highly robust with respect to the random choosing of different siblings.) After removing missing values in demographic and behavior measures, data of 8756 children were used in our analysis. The demographic characteristics of these participants are summarized in Table 1.

**Table 1:** The demographic characteristics of the ABCD participants.

Basic information									
Age (month)	Sex (Male/Female)	Body mass index	Parents income	Parents education	Puberty	Race (White/Black/Indian/Other)	Family conflict score	Parental monitoring score	Family psychiatric history (Fa/Mo)
119.02 $$\pm$$ 7.46	52%/48%	16.62 $$\pm$$ 2.75	7.24 $$\pm$$ 2.30	18.79 $$\pm$$ 4.17	1.61 $ 0.49	75%/21%/3%/1%	1.93 $$\pm$$ 1.82	4.39 $$\pm$$ 0.51	20%/31%
**Cognitive scores**									
Picture vocabulary test score	Flanker inhibitory control and attention test score	List sorting working memory score	Dimensional change card sort test score	Pattern comparison processing speed test score	Picture sequence memory test score	Oral reading recognition test score	Cognition fluid composite score	Crystallized composite score	Cognition total composite score
92.61 ± 9.45	86.44 $$\pm$$ 7.04	94.09 $$\pm$$ 9.05	91.70 $$\pm$$ 10.58	96.80 $$\pm$$ 12.00	88.18 $$\pm$$ 14.56	102.94 $$\pm$$ 12.08	84.55 $$\pm$$ 8.10	90.91 ± 6.88	86.36 ± 9.08
**Behavioral problems scores**									
Anxious/Depressed CBCL Syndrome Scale	Withdrawn/Depressed CBCL Syndrome Scale	Somatic Complaints CBCL Syndrome Scale	Social Problems CBCL Syndrome Scale	Thought CBCL Syndrome Scale	Attention Problems CBCL Syndrome Scale	Rule-breaking Behavior CBCL Syndrome Scale	Aggressive Behavior CBCL Syndrome Scale	Internalizing Problems CBCL Syndrome Scale	Externalizing Problems CBCL Syndrome Scale
2.38 $$\pm$$ 3.00	0.99 $$\pm$$ 1.63	1.41 $$\pm$$ 1.87	1.50 $$\pm$$ 2.16	1.53 $$\pm$$ 2.10	2.75 $$\pm$$ 3.43	1.11 $$\pm$$ 1.75	3.07 $$\pm$$ 4.30	4.93 $$\pm$$ 5.62	4.26 $$\pm$$ 5.86
TotProb CBCL Syndrome Scale	Depressive Problems CBCL DSM5 Scale	Anxiety Problems CBCL DSM5 sScale	Somatic Problems CBCL DSM5 Scale	ADHD CBCL DSM5 Scale	Oppositional Defiant Problems CBCL DSM5 Scale	Conduct Problems CBCL DSM5 Scale	Sluggish Cognitive Tempo CBCL Scale2007 Scale	Obsessive-Compulsive Problems CBCL Scale2007 Scale	Stress CBCL Scale2007 Scale
18.38 $$\pm$$ 17.98	1.21 $$\pm$$ 1.95	1.94 $$\pm$$ 2.34	1.04 $$\pm$$ 1.46	2.40 $$\pm$$ 2.87	1.66 $$\pm$$ 1.93	1.19 $$\pm$$ 2.25	0.51 $$\pm$$ 0.97	1.29 $$\pm$$ 1.73	2.74 $$\pm$$ 3.30

The data are shown as mean ± standard deviation or percentage.

### Family environment scores

Two family environment measures or scores were used in this paper, obtained from the ABCD Sum Scores Culture & Environment Youth (abcd_sscey01) based on youth surveys. The first was the family conflict score (fes_y_ss_fc_pr) estimated as the average of nine questions from the ABCD Parent Family Environment Scale-Family Conflict Subscale Modified from PhenX (FES, abcd_fes01), which reflects high conflict between family members including the parents and children^20^. A higher family conflict score indicates that there are more severe family conflicts in a child’s family. The second is the parental monitoring score (pmq_y_ss_mean), calculated as the average of the five questions from the ABCD Parental Monitoring Survey (pmq01), which reflects overall high parental monitoring behaviors^21^. The questions for these two scores and the ways they were calculated are included in Supplementary Material. The above two scores were collected at the ABCD baseline time, and the participants were followed up 1 year after the baseline time.

### Cognitive scores

Cognitive function was assessed by the ABCD Youth NIH TB Summary Scores (abcd_tbss01) which consists of ten validated and reliable psychometric test scores: Picture Vocabulary Test Score (nihtbx_picvocab); Flanker Inhibitory Control and Attention Test Score (nihtbx_flanker); List Sorting Working Memory Score (nihtbx_list); Dimensional Change Card Sort Test Score (nihtbx_cardsort); Pattern Comparison Processing Speed Test Score (nihtbx_pattern); Picture Sequence Memory Test Score (nihtbx_picture); Oral Reading Recognition Test Score (nihtbx_reading); Cognition Fluid Composite Score (nihtbx_fluidcomp); Crystallized Composite Score (nihtbx_cryst); and Cognition Total Composite Score (nihtbx_totalcomp)^57,58^. A high score means higher cognitive function.

### Behavioral problems scores

Dimensional psychopathology and adaptive functioning in children were assessed by the ABCD CBCL scores (abcd_cbcls01) based on parent surveys^59^. It contains 20 empirically based syndrome scales related to behavioral problems: Anxious/Depressed CBCL Syndrome Scale (cbcl_scr_syn_anxdep); Withdrawn/Depressed CBCL Syndrome Scale (cbcl_scr_syn_withdep); Somatic Complaints CBCL Syndrome Scale (cbcl_scr_syn_somatic); Social Problems CBCL Syndrome Scale (cbcl_scr_syn_social); Thought CBCL Syndrome Scale (cbcl_scr_syn_thought); Attention Problems CBCL Syndrome Scale (cbcl_scr_syn_attention); Rule-Breaking Behavior CBCL Syndrome Scale (cbcl_scr_syn_rulebreak); Aggressive Behavior CBCL Syndrome Scale (cbcl_scr_syn_aggressive); Internalizing Problems CBCL Syndrome Scale (cbcl_scr_syn_internal); Externalizing Problems CBCL Syndrome Scale (cbcl_scr_syn_external); TotProb CBCL Syndrome Scale (cbcl_scr_syn_totprob); Depressive Problems CBCL DSM5 Scale (cbcl_scr_dsm5_depress); Anxiety Problems CBCL DSM5 Scale (cbcl_scr_dsm5_anxdisord); Somatic Problems CBCL DSM5 Scale (cbcl_scr_dsm5_somaticpr); ADHD CBCL DSM5 Scale (cbcl_scr_dsm5_adhd); Oppositional Defiant Problems CBCL DSM5 Scale (cbcl_scr_dsm5_opposit); Conduct Problems CBCL DSM5 Scale (cbcl_scr_dsm5_conduct); Sluggish Cognitive Tempo CBCL Scale2007 Scale (cbcl_scr_07_sct); Obsessive-Compulsive Problems CBCL Scale2007 Scale (cbcl_scr_07_ocd); and Stress CBCL Scale2007 Scale (cbcl_scr_07_stress). A high score indicates dimensional psychopathology and a more severe behavioral problem. All of the above 20 scores were collected at the ABCD baseline time and the participants were followed up 1 year after the baseline time.

More details of the behavior assessments used in our analysis are provided in Supplementary Material (Table S1) and also can be found at the ABCD website (https://abcdstudy.org/scientists/protocols/).

### Association analysis

A general linear model (GLM) was used to test the associations of the family environment scores (family conflict and parental monitoring) with the brain morphometric measurements. Although a linear mixed effect model was recommended by the ABCD and used in other studies^60,61^, in the analysis of vertex-wise brain imaging data, the linear mixed effect model was computationally infeasible. Instead, we selected one child per family to eliminate any possible effects of correlations in the observations if they were from the same family, and used a computationally feasible GLM to perform the vertex-level association analysis. A brain morphometry measurement or behavioral score was modeled as the dependent variable, and the family conflict score (or the parental monitoring score) and the nuisance covariates to be regressed out were modeled as fixed effects. The following variables were used as nuisance covariates of no interest: childrens’ age, sex, body mass index, puberty score, race (coded as three-column dummy variables) and parents’ income, number of years of education, historical mental health problems (obtained from the subscores of ABCD Family History Assessment-question 11), and the scanning site of the structural image, in line with previous studies^22,60,61^. The results were consistent when the covariate of family income was coded as a dummy variable. A *t*-statistic was obtained for each GLM to reflect the association of the family conflict score (or the parental monitoring score) with the dependent variable. False discovery rate at 0.05 level was used to correct for multiple comparisons across all vertices and both scores.

We also used a GLM to test the associations of the family environment scores with other behavioral measures (e.g., all scores that listed in Table S1) that are provided by the ABCD dataset, and with some neuroimaging measurements such as the total cortical area. In this analysis, the same nuisance covariates of no interest were used. A Bonferroni correction approach (*p* < 0.05) was used to adjust for multiple comparisons.

In all the above analyses, all brain measurements and behavioral variables were collected at the ABCD baseline time.

### Mediation analysis

A standard mediation analysis was performed using the Mediation Toolbox developed by Tor Wager’s group (https://github.com/canlab/MediationToolbox), which has been widely used in many neuroimaging studies^62–64^. A standard three-variable path model was used here^65^, with the detailed methodology description in Supplementary Material of ref. ^62^. Briefly, mediation analysis tests whether the association between two variables can be explained by a third variable (the mediator). The hypothesis tested here was whether the behavioral problems total score (TotProb CBCL Syndrome Scale) mediated the association between surface areas of the brain and the family conflict score/parental monitoring score. Confounding variables as in the association analysis were regressed out in the mediation model. The significance of the mediation was estimated by the bias-corrected bootstrap approach (with 10,000 random samplings). In this analysis, all brain measurements and behavioral variables were collected at the ABCD baseline time.

### Longitudinal association analysis

For the above-mentioned measurements, it was possible to perform a longitudinal analysis using the family conflict scores, or parental monitoring scores, and the behavioral problems total scores that were obtained in the follow-up 1 year after the baseline time. A classic two-wave CLPM based on structural equation modeling was implemented to investigate the longitudinal associations between the family conflict scores or parental monitoring scores and the behavioral problems total scores^66,67^. Specifically, the relative strength of the cross-lagged relationships between the behavioral problems total scores and the family conflict scores or the parental monitoring scores all measured at the baseline time with the 1-year follow-up were evaluated by a cross-lagged panel structural model implemented by Mplus (version 7.4)^68^. Effects in both directions were considered. The model was estimated by using maximum likelihood estimation with robust standard errors that also takes clustering of cases into account. The standardized regression coefficients and standard errors are reported throughout. Confounding variables as in the association analysis were regressed out in the CLPM analysis.

### Reporting summary

Further information on research design is available in the Nature Research Reporting Summary linked to this article.

## Supplementary information

Supplementary Information

Reporting Summary

## Data Availability

Neuroimaging and behavioral data from ABCD dataset are obtained from https://nda.nih.gov/abcd with the approval of the ABCD consortium. A reporting summary for this article is available as a Supplementary Information file.

## Code availability

The data preprocessing software FreeSurfer v6.0 can be obtained from https://surfer.nmr.mgh.harvard.edu/. Qoala-T v1.2 for automatic quality control can be obtained from: https://github.com/Qoala-T/QC. Matlab 2019a was used for the brain and behavioral association analysis. The scripts can be downloaded from a GitHub repository at: https://github.com/weikanggong/ABCD_family_environment. Software for the mediation analysis can be obtained from https://github.com/canlab/MediationToolbox. Software for the CLPM analysis can be obtained from https://www.statmodel.com.
