# Supplementary material for: Brain structure is linked to the association between family environment and behavioral problems in children in the ABCD study
Source: Nat Commun. 2021 Jun 18;12:3769. doi: 10.1038/s41467-021-23994-0 (PMC8213719; doi:10.1038/s41467-021-23994-0)

Supplementary Information

Brain structure is linked to the association between family environment and behavioral problems in children in the ABCD study

Nature Communications (2021)

Weikang Gong [1,#], Edmund T. Rolls[2,3,4,5,#], Jingnan Du[2,3,#], Jianfeng Feng[2,3,4,6], Wei Cheng[2,3,6,*]

1. Centre for Functional MRI of the Brain (FMRIB), Nuffield Department of Clinical Neurosciences, Wellcome Centre for Integrative Neuroimaging, University of Oxford, Oxford, OX3 9DU, UK.
2. Institute of Science and Technology for Brain-inspired Intelligence, Fudan University, Shanghai, 200433, China.
3. Key Laboratory of Computational Neuroscience and Brain-Inspired Intelligence, Fudan University, Ministry of Education, Shanghai, 200433, China.
4. Department of Computer Science, University of Warwick, Coventry, CV4 7AL, UK.
5. Oxford Centre for Computational Neuroscience, Oxford, UK.
6. Fudan ISTBI—ZJNU Algorithm Centre for Brain-inspired Intelligence, Zhejiang Normal University, Jinhua, China.

# These authors contributed equally to this work.

* Corresponding author
Wei Cheng, Institute of Science and Technology for Brain-inspired Intelligence, Shanghai, 200433, China, E-mail address: wcheng@fudan.edu.cn.

Questions used in the Family Conflict score and Parental Monitoring scores and the methods by which they were calculated.

Family Conflict score
fes_youth_q1: We fight a lot in our family.
fes_youth_q2: Family members rarely become openly angry.
fes_youth_q3: Family members sometimes get so angry they throw things.
fes_youth_q4: Family members hardly ever lose their tempers.
fes_youth_q5: Family members often criticize each other.
fes_youth_q6: Family members sometimes hit each other.
fes_youth_q7: If there is a disagreement in our family, we try hard to smooth things over and keep the peace.
fes_youth_q8: Family members often try to one-up or outdo each other.
fes_youth_q9: In our family, we believe you don't ever get anywhere by raising your voice.

Family conflict score = (fes_youth_q1 + fes_youth_q2 + fes_youth_q3 + fes_youth_q4 + fes_youth_q5 + fes_youth_q6 + fes_youth_q7 + fes_youth_q8 + fes_youth_q9) * fes_y_ss_fc_nt/ fes_y_ss_fc_na.
where fes_y_ss_fc_nt is the number of total questions and fes_y_ss_fc_nt is the number of questions answered.

Parental Monitoring score
parent_monitor_q1_y: How often do your parents/guardians know where you are?
parent_monitor_q2_y: How often do your parents know who you are with when you are not at school and away from home?
parent_monitor_q3_y: If you are at home when your parents or guardians are not, how often do you know how to get in touch with them?
parent_monitor_q4_y: How often do you talk to your mom/dad or guardian about your plans for the coming day, such as your plans about what will happen at school or what you are going to do with friends?
parent_monitor_q5_y: In an average week, how many times do you and your parents/guardians, eat dinner together?

Parental monitoring score = Mean (parent_monitor_q1_y + parent_monitor_q2_y + parent_monitor_q3_y + parent_monitor_q4_y parent_monitor_q5_y)/5

Supplementary Table 1. All measures used in this study.

Measure	NDA Short Name	Description
**Physical and Mental Health**		
ABCD Youth Anthropometrics Modified From PhenX (ANT)	abcd_ant01	Height, weight, waist circumference
Adult Self Report Scores	abcd_asrs01	Adult Self Report summary scores
ABCD Youth Behavioral Inhibition/Behavioral Approach System Scales Modified from PhenX (BIS/BAS)	abcd_bisbas01	Inhibition and reward seeking
ABCD Parent Child Behavior Checklist Raw Scores Aseba (CBCL)	abcd_cbcl01	Dimensional psychopathology, adaptive functioning
Child Behavior Check List Scores	abcd_cbcls01	Child Behavior Check List summary scores
ABCD Youth Edinburgh Handedness Inventory Short Form (EHIS)	abcd_ehis01	Handedness, laterality quotient
ABCD Youth Diagnostic Interview for DSM-5 (KSADS-5)	abcd_ksad501	Mental health diagnosis - youth questions
ABCD Sum Scores Mental Health Parent	abcd_mhp01	Mental Health summary scores - parent surveys
ABCD Parent Medical History Questionnaire (MHX)	abcd_mx01	Medical history and health services utilization
ABCD Parent Ohio State Traumatic Brain Injury Screen-Short Modified (OTBI)	abcd_otbi01	Traumatic brain injury of youth
ABCD Parent General Behavior Inventory-Mania (PGBI)	abcd_pgbi01	Subsyndromal mania
ABCD Parent Pubertal Development Scale and Menstrual Cycle Survey History (PDMS)	abcd_ppdms01	Pubertal stage and menstrual phase (for postmenarcheal girls) - parent survey
ABCD Parent Sports and Activities Involvement Questionnaire (SAIQ)	abcd_saiq01	Involvement in sports, music and hobbies, TBI risk
ABCD Parent Sleep Disturbance Scale for Children (SDS)	abcd_sds01	Sleep and sleep disorders
ABCD Youth Screen Time Survey (STQ)	abcd_stq01	Screen time utilization - youth
ABCD Youth Snellen Vision Screener (SVS)	abcd_svs01	Vision screening
ABCD Sum Scores Traumatic Brain Injury	abcd_tbi01	Traumatic brain injury of youth summary scores
UPPS-P for Children Short Form (ABCD-version)	abcd_upps01	Impulsivity
ABCD Youth Diagnostic Interview for DSM-5 Background Items (KSADS-5)	abcd_yksad01	School, sexual orientation
ABCD Youth Pubertal Development Scale and Menstrual Cycle Survey History (PDMS)	abcd_ypdms01	Pubertal stage and menstrual phase (for postmenarcheal girls) - youth survey
ABCD Youth Risk Behavior Survey Exercise Physical Activity (YRB)	abcd_yrb01	Physical exercise
ABCD Youth Resilience Scale (YRS)	abcd_ysr01	Resilience (religiosity, friends)
ABCD Developmental History Questionnaire	dhx01	Prenatal exposure before and during pregnancy - medications, drugs alcohol, tobacco
ABCD Parent Diagnostic Interview for DSM-5 Background Items Full (KSADS-5)	dibf01	School, family, social relations
ABCD Family History Assessment Part 1	fhxp101	Family history of psychopathology and substance use (for biological or adoptive parent)
ABCD Family History Assessment Part 2	fhxp201	Family history of psychopathology and substance use (for biological or adoptive parent)
ABCD Parent Medications Survey Inventory Modified from PhenX (PMP)	medsy01	Medications taken in the last two weeks
ABCD Parent Adult Self Report Raw Scores Aseba (ASR)	pasr01	Parent dimensional psychopathology
ABCD Parent Demographics Survey?	pdem01	Demographics, race, gender, family structure, SES, education, occupation (includes Native American Acculturation Scale)
ABCD Prodromal Psychosis Scale	pps01	Prodromal psychosis levels
ABCD Parent Screen Time Survey (STQ)	stq01	Screen time utilization - parent
**Substance Use**		
ABCD Parent Community Risk and Protective Factors (CRPF)	abcd_crpf01	Beliefs about drug availability (alcohol, nicotine, marijuana, ?other? drugs) along with questions about access and exposure to medical marijuana
ABCD Youth Participant Last Use Survey Day 1 2 3 4 (PLUS)	abcd_plus01	Tobacco/caffeine/medication usage in the last 24 hours - youth answers
ABCD Youth Substance Use Interview	abcd_ysu01	Measures included in Substance Use Interview are described below
ABCD Parent Participant Last Use Survey Day 2 3 4 (PLUS)	plus01	Tobacco/caffeine/medication usage in the last 24 hours - parent answers
ABCD Parental Rules on Substance Use	prq01	Parental substance use approval and rules
**Culture and Environment**		
ABCD Youth Family Environment Scale-Family Conflict Subscale Modified from PhenX (FES)	abcd_fes01	Family dynamics, cohesion, expressiveness, conflict
ABCD Parent Multi-Group Ethnic Identity-Revised Survey (MEIM)	abcd_meim01	Cultural affiliation
ABCD Youth Neighborhood Safety/Crime Survey Modified from PhenX (NSC)	abcd_nsc01	Neighborhood risk and protective factors, crime
ABCD Parent Neighborhood Safety/Crime Survey Modified from PhenX (NSC)	abcd_pnsc01	Neighborhood risk and protective factors, crime
Youth Prosocial Behavior Survey	abcd_psb01	Resilience
ABCD Sum Scores Culture & Environment Parent	abcd_sscep01	Culture and environment summary scores - parent surveys
ABCD Sum Scores Culture & Environment Youth	abcd_sscey01	Culture and environment summary scores - youth surveys
ABCD Parent Vancouver Index of Acculturation-Short Survey (VIA)	abcd_via01	Acculturation
ABCD Children's Report of Parental Behavioral Inventory	crpbi01	Environment- family and religion
ABCD Parent Family Environment Scale-Family Conflict Subscale Modified from PhenX (FES)	fes01	Family dynamics, cohesion, expressiveness, conflict
ABCD Parent Mexican American Cultural Values Scale Modified (MACV)	macv01	Familism, religion, independence, self-reliance
ABCD Parent Acculturation Survey Modified from PhenX (ACC)	pacc01	Cultural factors
ABCD Parental Monitoring Survey	pmq01	Parental monitoring and supervision
Parent Prosocial Behavior Survey	psb01	Resilience
ABCD School Risk and Protective Factors Survey	srpf01	School risk and protective factors
ABCD Youth Acculturation Survey Modified from PhenX (ACC)	yacc01	Cultural factors
**Neurocognition**		
ABCD Pearson Scores	absd_ps01	Measures included in Pearson Scores are described below
ABCD Youth NIH TB Summary Scores	abcd_tbss01	Measures included in NIH TB Summary Scores are described below
ABCD TBX Demo	abcddemo01	Demographics - age only
ABCD Little Man Task	lmtp201	Visuospatial processing flexibility, attention

Others		
ABCD Youth Hair Sample	abcd_hers01	Information collected by RAs at the time of collecting ~100 strands of hair for metabolites indicating substance use in past month or longer (e.g. time of day of collection)
ABCD Sum Scores Mental Health Youth	Sum_Scores_mhy01	Mental Health summary scores - youth surveys
ABCD MRI Behavior MID	abcd_mid01	Behavioral performance measures for MID task fMRI
ABCD Youth Monetary Incentive Delay (MID) Task Survey Post Scan Questionnaire	abcd_monet01	Ratings of mood when viewing the different cues and receiving the different outcomes during the MID task to determine the effectiveness and value of wins and losses
MR Findings	abcd_mrfindings01	Neuroradiology reports - scores, hydrocephalus and herniation
ABCD RA Scanning Checklist and Notes	abcd_ra01	Checklist used by site coordinators/research assistants prior to and during scanning sessions
Residential History Derived Scores	abcd_rhds01	Environmental risk
ABCD Screener	abcd_screen01	Eligibility screener and screener risk measures
ABCD MRI Behavior SST	abcd_sst01	Behavioral performance measures for SST task fMRI
ABCD Youth Genetic Saliva (RUCDR)	abcd_ygs01	Information collected by RAs at the time of saliva collection for genetics studies (e.g., time of day of collection)
ABCD Youth Pre Scan Questionnaire 1	abcd_ypre101	Current mood ratings before completing MRI scan
ABCD Youth Post Scan Questionnaire 1	abcd_ypsq101	Current mood ratings after completing MRI scan
ABCD Youth Toxicology Test	abcd_ytt01	Past day drug use - Oral Fluid Drager
ACS Post Stratification Weights	acspsw01	ACS post stratification weights for demographic and socioeconomic measures, and family relationships table
ABCD Youth Genetic Blood (RUCDR)	biocf01	Information collected by RAs at the time of venipuncture blood collection for genetics studies (e.g., time of day of collection)
Processed MRI Data (used for minimally processed data)	fmriresults01	Index of minimally processed data
ABCD Pubertal Hormone Saliva	sph01	Information collected by RAs at the time of collecting oral fluid to indicate current estradiol, testosterone, and DHEA levels (e.g., time of day of collection)

Supplementary Table 2. Correlation of the family conflict and the parental monitoring score with the lifetime mental disorder diagnoses based on the KSADS assessment (abcd_ksad501).

KSADS Items	Family Conflict		Parental Monitoring	
	t value	p value	t value	p value
Diagnosis - Bipolar I Disorder, current episode manic (F31.1x)	3.070	2.15E-03	-2.231	2.57E-02
Diagnosis - Bipolar I Disorder, current episode depressed, F31.3x	5.987	2.21E-09	-3.147	1.65E-03
Diagnosis - Bipolar I Disorder, currently hypomanic F31.0	2.355	1.85E-02	-3.580	3.45E-04
Diagnosis - Bipolar I Disorder, most recent past episode manic (F31.1x)	5.469	4.63E-08	-2.896	3.79E-03
Diagnosis - Bipolar I Disorder, most recent past episode depressed (F31.1.3x)	1.913	5.57E-02	-0.288	7.73E-01
Diagnosis - Bipolar II Disorder, currently hypomanic F31.81	1.410	1.59E-01	-1.323	1.86E-01
Diagnosis - Bipolar II Disorder, currently depressed F31.81	4.942	7.85E-07	-0.898	3.69E-01
Diagnosis - Bipolar II Disorder, most recent past hypomanic F31.81	6.746	1.61E-11	-2.927	3.43E-03
Diagnosis - Unspecified Bipolar and Related Disorder, current (F31.9)	5.490	4.12E-08	-3.753	1.76E-04
Diagnosis - Unspecified Bipolar and Related Disorder, PAST (F31.9)	7.820	5.88E-15	-2.673	7.54E-03
Diagnosis - Major Depressive Disorder Present	4.978	6.52E-07	-4.683	2.87E-06
Diagnosis - Major Depressive Disorder, Current, in Partial Remission (F32.4)	2.593	9.52E-03	-1.738	8.22E-02
Diagnosis - Major Depressive Disorder, Past (F32.9)	7.066	1.71E-12	-6.191	6.24E-10
Diagnosis - Persistent Depressive Disorder (Dysthymia) PAST F34.1	2.535	1.12E-02	-0.812	4.17E-01
Diagnosis - Unspecified Depressive Disorder Current (F32.9)	1.918	5.52E-02	-0.733	4.64E-01
Diagnosis - Unspecified Depressive Disorder PAST (F32.9)	5.659	1.57E-08	-4.768	1.88E-06
Diagnosis - Social Anxiety Disorder (F40.10) PRESENT	3.278	1.05E-03	-3.033	2.43E-03
Diagnosis - Social Anxiety Disorder (F40.10) PAST	1.312	1.89E-01	-2.612	9.02E-03
Diagnosis - Generalized Anxiety Disorder Present (F41.1)	2.713	6.68E-03	-2.342	1.92E-02
Diagnosis - Generalized Anxiety Disorder Past (F41.1)	3.899	9.74E-05	-1.082	2.79E-01
Diagnosis - Other Specified Anxiety Disorder (Social Anxiety Disorder, impairment, does not meet minimum duration), PAST, F41.8	3.111	1.87E-03	-2.920	3.51E-03
Diagnosis - Other Specified Anxiety Disorder (Generalized Anxiety Disorder, impairment, does not meet minimum duration) F41.8	2.223	2.62E-02	-3.448	5.67E-04
Diagnosis - Other Specified Anxiety Disorder (Generalized Anxiety Disorder, impairment, does not meet minimum duration), PAST, F41.8	4.551	5.39E-06	-2.888	3.88E-03
Diagnosis - SelfInjuriousBehaviorwithoutsuicidalintentPresent	8.213	2.22E-16	-9.255	<1.0E-10
Diagnosis - SuicidalideationPassivePresent	8.082	6.66E-16	-8.312	1.11E-16
Diagnosis - SuicidalideationActivenonspecificPresent	9.495	<1.0E-10	-7.729	1.20E-14
Diagnosis - SuicidalideationActivemethodPresent	7.306	2.98E-13	-7.698	1.52E-14
Diagnosis - SuicidalideationActiveintentPresent	5.122	3.09E-07	-4.918	8.89E-07
Diagnosis - SuicidalideationActiveplanPresent	4.090	4.34E-05	-5.881	4.23E-09
Diagnosis - PreparatoryActionstowardimminentSuicidalbehaviorPresent	3.295	9.89E-04	-4.829	1.40E-06
Diagnosis - InterruptedAttemptPresent	1.612	1.07E-01	-2.583	9.82E-03
Diagnosis - AbortedAttemptPresent	3.084	2.05E-03	-2.443	1.46E-02
Diagnosis - SuicideAttemptPresent	1.251	2.11E-01	-1.690	9.10E-02
Diagnosis - SelfInjuriousBehaviorwithoutsuicidalintentPast	6.923	4.73E-12	-7.803	6.66E-15
Diagnosis - SuicidalideationPassivePast	11.616	<1.0E-10	-8.127	4.44E-16
Diagnosis - SuicidalideationActivenonspecificPast	9.806	<1.0E-10	-8.806	<1.0E-10
Diagnosis - SuicidalideationActivemethodPast	5.690	1.31E-08	-4.593	4.42E-06
Diagnosis - SuicidalideationActiveintentPast	4.675	2.98E-06	-2.855	4.31E-03
Diagnosis - SuicidalideationActiveplanPast	0.173	8.62E-01	-3.116	1.84E-03
Diagnosis - PreparatoryActionstowardimminentSuicidalbehaviorPast	3.824	1.32E-04	-3.112	1.86E-03
Diagnosis - InterruptedAttemptPast	1.529	1.26E-01	-3.150	1.64E-03
Diagnosis - AbortedAttemptPast	2.419	1.56E-02	-4.424	9.81E-06
Diagnosis - SuicideAttemptPast	5.612	2.06E-08	-4.807	1.56E-06
Diagnosis - SLEEP PROBLEMS, Present	10.663	<1.0E-10	-8.428	<1.0E-10
Diagnosis - SLEEP PROBLEMS, Past	10.470	<1.0E-10	-6.909	5.19E-12

Supplementary Table 3. The longitudinal association between childrens' behavioral problems subscores (TotProb CBCL Syndrome Sub-Scales) and the family conflict score using structural equation modelling. Beta 1 and the corresponding p-value 1 indicate the association between the family conflict score and behavioral problems subscores both at baseline. Beta 2 and the corresponding p-value 2 indicate the longitudinal association between the family conflict scores at baseline and the behavioral problems subscores at the one-year follow-up. Beta 3 and the corresponding p-value 3 indicate the longitudinal association between the behavioral problems subscores at baseline and the family conflict score at the one-year follow-up. Beta 4 and the corresponding p-value 4 indicate the association between the family conflict score and behavioral problems subscores both at the one-year follow-up. A star indicates a significant effect after FDR correction (p<0.05).

**Behavioral problems subscores**	**Beta 1**	**p-value 1**	**Beta 2**	**p-value 2**	**Beta 3**	**p-value 3**	**Beta 4**	**p-value 4**
cbcl_scr_07_ocd_r	0.035	0.038	0.006	0.064	0.051	0.001*	0.036	0.035
cbcl_scr_07_sct_r	0.062	<0.001*	0.012	0.354	0.042	0.005*	0.05	0.002*
cbcl_scr_07_stress_r	0.093	<0.001*	0.01	0.442	0.083	<0.001*	0.093	<0.001*
cbcl_scr_dsm5_adhd_r	0.093	<0.001*	0.012	0.278	0.088	<0.001*	0.072	<0.001*
cbcl_scr_dsm5_anxdisord_r	0.025	0.125	<0.001	0.97	0.042	0.006*	0.025	0.135
cbcl_scr_dsm5_conduct_r	0.142	<0.001*	0.025	0.061	0.11	<0.001*	0.111	<0.001*
cbcl_scr_dsm5_depress_r	0.084	<0.001*	0.042	0.003*	0.079	<0.001*	0.071	<0.001*
cbcl_scr_dsm5_opposit_r	0.145	<0.001*	0.026	0.031*	0.1	<0.001*	0.108	<0.001*
cbcl_scr_dsm5_somaticpr_r	0.018	0.255	0.01	0.475	0.006	0.657	0.016	0.346
cbcl_scr_syn_aggressive_r	0.143	<0.001*	0.021	0.101	0.102	<0.001*	0.111	<0.001*
cbcl_scr_syn_anxdep_r	0.037	0.021*	0.009	0.464	0.059	<0.001*	0.046	0.007*
cbcl_scr_syn_attention_r	0.103	<0.001*	0.001	0.945	0.087	<0.001*	0.069	<0.001*
cbcl_scr_syn_external_r	0.148	<0.001*	0.019	0.132	0.11	<0.001*	0.115	<0.001*
cbcl_scr_syn_internal_r	0.055	0.001*	0.017	0.171	0.059	<0.001*	0.049	0.004*
cbcl_scr_syn_rulebreak_r	0.131	<0.001*	0.024	0.067	0.107	<0.001*	0.098	<0.001*
cbcl_scr_syn_social_r	0.073	<0.001*	0.029	0.031*	0.078	<0.001*	0.073	<0.001*
cbcl_scr_syn_somatic_r	0.028	0.086	0.011	0.434	0.024	0.103	0.022	0.189
cbcl_scr_syn_thought_r	0.086	<0.001*	0.015	0.293	0.078	<0.001*	0.047	0.006*
cbcl_scr_syn_totprob_r	0.118	<0.001*	0.014	0.225	0.101	<0.001*	0.087	<0.001*
cbcl_scr_syn_withdep_r	0.08	<0.001*	0.034	0.019*	0.056	<0.001*	0.051	0.002*

Supplementary Table 4. The longitudinal association between the childrens' behavioral problems subscores (TotProb CBCL Syndrome Sub-Scales) and the parental monitoring score using structural equation modelling. Beta 1 and the corresponding p-value 1 indicate the association between the parental monitoring score and the behavioral problems subscores both at baseline. Beta 2 and the corresponding p-value 2 indicates the longitudinal association between the parental monitoring score at baseline and the behavioral problems subscores at the one-year follow-up. Beta 3 and the corresponding p-value 3 indicate the longitudinal association between the behavioral problems subscores at baseline and the parental monitoring score at the one-year follow-up. Beta 4 and the corresponding p-value 4 indicate the association between the behavioral problems subscores and the parental monitoring scores both at the one-year follow-up. A star indicates a significant effect after FDR correction (p<0.05).

**Parental monitoring score**	**beta 1**	**p-value 1**	**beta 2**	**p-value 2**	**beta 3**	**p-value 3**	**beta 4**	**P-value 4**
cbcl_scr_07_ocd_r	-0.014	0.016*	-0.026	0.043	-0.047	0.003*	-0.029	0.094
cbcl_scr_07_sct_r	-0.093	<0.001*	-0.049	<0.001*	-0.089	<0.001*	-0.065	<0.001*
cbcl_scr_07_stress_r	-0.102	<0.001*	-0.031	0.013*	-0.082	<0.001*	-0.058	0.001*
cbcl_scr_dsm5_adhd_r	-0.114	<0.001*	-0.022	0.051	-0.115	<0.001*	-0.038	0.03*
cbcl_scr_dsm5_anxdisord_r	-0.068	<0.001*	-0.032	0.01*	-0.046	0.002*	-0.033	0.052
cbcl_scr_dsm5_conduct_r	-0.122	<0.001*	-0.015	0.259	-0.041	0.02*	-0.062	0.002*
cbcl_scr_dsm5_depress_r	-0.082	<0.001*	-0.048	0.001*	-0.077	<0.001*	-0.077	<0.001*
cbcl_scr_dsm5_opposit_r	-0.08	<0.001*	-0.031	0.009*	-0.072	<0.001*	-0.049	0.004*
cbcl_scr_dsm5_somaticpr_r	0.006	0.698	-0.014	0.365	-0.014	0.32	-0.028	0.082
cbcl_scr_syn_aggressive_r	-0.088	<0.001*	-0.021	0.069	-0.066	<0.001*	-0.038	0.027*
cbcl_scr_syn_anxdep_r	-0.067	<0.001*	-0.035	0.003*	-0.046	0.003*	-0.035	0.044
cbcl_scr_syn_attention_r	-0.143	<0.001*	-0.035	0.002*	-0.139	<0.001*	-0.055	0.003*
cbcl_scr_syn_external_r	-0.101	<0.001*	-0.019	0.118	-0.067	<0.001*	-0.047	0.01*
cbcl_scr_syn_internal_r	-0.078	<0.001*	-0.039	0.002*	-0.053	<0.001*	-0.066	<0.001*
cbcl_scr_syn_rulebreak_r	-0.112	<0.001*	-0.019	0.168	-0.054	0.002*	-0.059	0.003*
cbcl_scr_syn_social_r	-0.107	<0.001*	-0.029	0.03*	-0.091	<0.001*	-0.039	0.03*
cbcl_scr_syn_somatic_r	-0.009	0.586	-0.019	0.179	-0.022	0.112	-0.04	0.013*
cbcl_scr_syn_thought_r	-0.093	<0.001*	-0.023	0.093	-0.066	<0.001*	-0.05	0.006*
cbcl_scr_syn_totprob_r	-0.12	<0.001*	-0.031	0.007*	-0.094	<0.001*	-0.061	0.001*
cbcl_scr_syn_withdep_r	-0.125	<0.001*	-0.048	0.001*	-0.064	<0.001*	-0.097	<0.001*

Supplementary Table 5. The mediation by the 19 behavioral problems subscores for the association between cortical areas in the children and the family conflict. The analysis is analogous to that shown in Fig. 3C, but here is for subscores, instead of behavioral problems total scores. The paths correspond to those shown in Fig. 3C. All subscores except cbcl_scr_dsm5_somaticpr were found to mediate the relation between cortical areas and family conflict in this analysis after FDR correction (p<0.05).

Behavioral problems subscores	Path A		Path B		Path C		Path C’		Path AB	
	beta	p-value	beta	p-value	beta	p-value	beta	p-value	beta	p-value
cbcl_scr_syn_anxdep	-0.044	3.18E-05	0.054	5.01E-07	-0.070	4.40E-11	-0.068	1.90E-10	-0.002	1.53E-03
cbcl_scr_syn_withdep	-0.056	2.06E-07	0.090	<1.0E-10	-0.070	4.40E-11	-0.065	8.31E-10	-0.005	1.07E-05
cbcl_scr_syn_somatic	-0.030	4.79E-03	0.046	1.86E-05	-0.070	4.40E-11	-0.069	1.02E-10	-0.001	2.08E-02
cbcl_scr_syn_social	-0.070	4.60E-11	0.096	<1.0E-10	-0.070	4.40E-11	-0.064	2.31E-09	-0.007	1.23E-07
cbcl_scr_syn_thought	-0.049	4.23E-06	0.070	6.68E-11	-0.070	4.40E-11	-0.067	3.44E-10	-0.003	1.90E-04
cbcl_scr_syn_attention	-0.088	2.22E-16	0.094	<1.0E-10	-0.070	4.40E-11	-0.062	5.78E-09	-0.008	2.12E-09
cbcl_scr_synulebreak	-0.073	8.76E-12	0.117	<1.0E-10	-0.070	4.40E-11	-0.062	5.93E-09	-0.009	7.42E-09
cbcl_scr_syn_aggressive	-0.085	2.44E-15	0.135	<1.0E-10	-0.070	4.40E-11	-0.059	2.79E-08	-0.011	1.97E-11
cbcl_scr_syn_internal	-0.052	9.21E-07	0.074	5.30E-12	-0.070	4.40E-11	-0.067	4.45E-10	-0.004	7.09E-05
cbcl_scr_syn_external	-0.086	6.66E-16	0.138	<1.0E-10	-0.070	4.40E-11	-0.059	3.56E-08	-0.012	7.72E-12
cbcl_scr_dsm5_depress	-0.058	7.02E-08	0.092	<1.0E-10	-0.070	4.40E-11	-0.065	1.00E-09	-0.005	5.24E-06
cbcl_scr_dsm5_anxdisord	-0.048	6.24E-06	0.045	2.05E-05	-0.070	4.40E-11	-0.068	1.73E-10	-0.002	2.21E-03
cbcl_scr_dsm5_somaticpr	-0.023	3.11E-02	0.035	9.84E-04	-0.070	4.40E-11	-0.070	7.22E-11	-0.001	8.03E-02
cbcl_scr_dsm5_adhd	-0.083	6.22E-15	0.089	<1.0E-10	-0.070	4.40E-11	-0.063	3.61E-09	-0.007	1.41E-08
cbcl_scr_dsm5_opposit	-0.078	1.95E-13	0.129	<1.0E-10	-0.070	4.40E-11	-0.060	1.42E-08	-0.010	3.38E-10
cbcl_scr_dsm5_conduct	-0.077	7.29E-13	0.128	<1.0E-10	-0.070	4.40E-11	-0.061	1.19E-08	-0.010	7.53E-10
cbcl_scr_07_sct	-0.039	2.97E-04	0.053	7.77E-07	-0.070	4.40E-11	-0.068	1.52E-10	-0.002	3.96E-03
cbcl_scr_07_ocd	-0.031	3.31E-03	0.042	6.98E-05	-0.070	4.40E-11	-0.069	9.96E-11	-0.001	2.05E-02
cbcl_scr_07_stress	-0.076	1.08E-12	0.092	<1.0E-10	-0.070	4.40E-11	-0.063	2.83E-09	-0.007	4.19E-08

Supplementary Table 6. The mediation by the 19 behavioral problems subscores for the association between cortical areas in the children and the parental monitoring. The analysis is analogous to that shown in Fig. 3D, but here is for subscores, instead of behavioral problems total scores. The paths correspond to those shown in Fig. 3D. All subscores except cbcl_scr_syn_somatic and cbcl_scr_dsm5_somaticpr were found to medite the relation between cortical areas and family conflict in this analysis after FDR correction (p<0.05).

Behavioral problems subscores	Path A		Path B		Path C		Path C’		Path AB	
	beta	p-value	beta	p-value	beta	p-value	beta	p-value	beta	p-value
cbcl_scr_syn_anxdep	-0.044	4.03E-05	-0.052	9.75E-07	0.068	2.02E-10	0.066	7.87E-10	0.002	1.88E-03
cbcl_scr_syn_withdep	-0.056	1.62E-07	-0.116	<1.0E-10	0.068	2.02E-10	0.061	7.31E-09	0.006	2.53E-06
cbcl_scr_syn_somatic	-0.031	4.21E-03	-0.024	2.62E-02	0.068	2.02E-10	0.067	3.17E-10	0.001	9.05E-02
cbcl_scr_syn_social	-0.068	1.54E-10	-0.091	<1.0E-10	0.068	2.02E-10	0.062	7.12E-09	0.006	3.36E-07
cbcl_scr_syn_thought	-0.050	2.57E-06	-0.069	1.14E-10	0.068	2.02E-10	0.064	1.51E-09	0.003	1.62E-04
cbcl_scr_syn_attention	-0.086	1.11E-15	-0.120	<1.0E-10	0.068	2.02E-10	0.058	5.96E-08	0.010	7.15E-11
cbcl_scr_synulebreak	-0.072	1.86E-11	-0.075	1.58E-12	0.068	2.02E-10	0.063	4.77E-09	0.005	1.26E-06
cbcl_scr_syn_aggressive	-0.079	1.52E-13	-0.065	1.33E-09	0.068	2.02E-10	0.063	4.21E-09	0.005	3.10E-06
cbcl_scr_syn_internal	-0.052	9.32E-07	-0.073	8.69E-12	0.068	2.02E-10	0.064	1.85E-09	0.004	7.57E-05
cbcl_scr_syn_external	-0.082	2.18E-14	-0.072	1.35E-11	0.068	2.02E-10	0.062	6.48E-09	0.006	4.57E-07
cbcl_scr_dsm5_depress	-0.056	1.59E-07	-0.080	4.82E-14	0.068	2.02E-10	0.063	2.71E-09	0.005	1.87E-05
cbcl_scr_dsm5_anxdisord	-0.049	5.52E-06	-0.047	9.92E-06	0.068	2.02E-10	0.066	8.01E-10	0.002	1.75E-03
cbcl_scr_dsm5_somaticpr	-0.024	2.76E-02	-0.011	2.93E-01	0.068	2.02E-10	0.068	2.40E-10	0.000	3.80E-01
cbcl_scr_dsm5_adhd	-0.080	5.88E-14	-0.092	<1.0E-10	0.068	2.02E-10	0.061	1.38E-08	0.007	1.68E-08
cbcl_scr_dsm5_opposit	-0.075	2.60E-12	-0.065	1.03E-09	0.068	2.02E-10	0.063	3.64E-09	0.005	4.73E-06
cbcl_scr_dsm5_conduct	-0.073	9.88E-12	-0.081	2.46E-14	0.068	2.02E-10	0.062	6.25E-09	0.006	4.24E-07
cbcl_scr_07_sct	-0.038	3.29E-04	-0.085	2.00E-15	0.068	2.02E-10	0.065	1.26E-09	0.003	1.15E-03
cbcl_scr_07_ocd	-0.033	1.80E-03	-0.043	6.72E-05	0.068	2.02E-10	0.067	4.70E-10	0.001	1.59E-02
cbcl_scr_07_stress	-0.075	2.18E-12	-0.075	2.02E-12	0.068	2.02E-10	0.062	5.45E-09	0.006	7.49E-07

Supplementary Figure 1. The brain regions with their cortical areas correlated with the family conflict score (left) and parental monitoring score (right). Results with two random choices of siblings are presented.

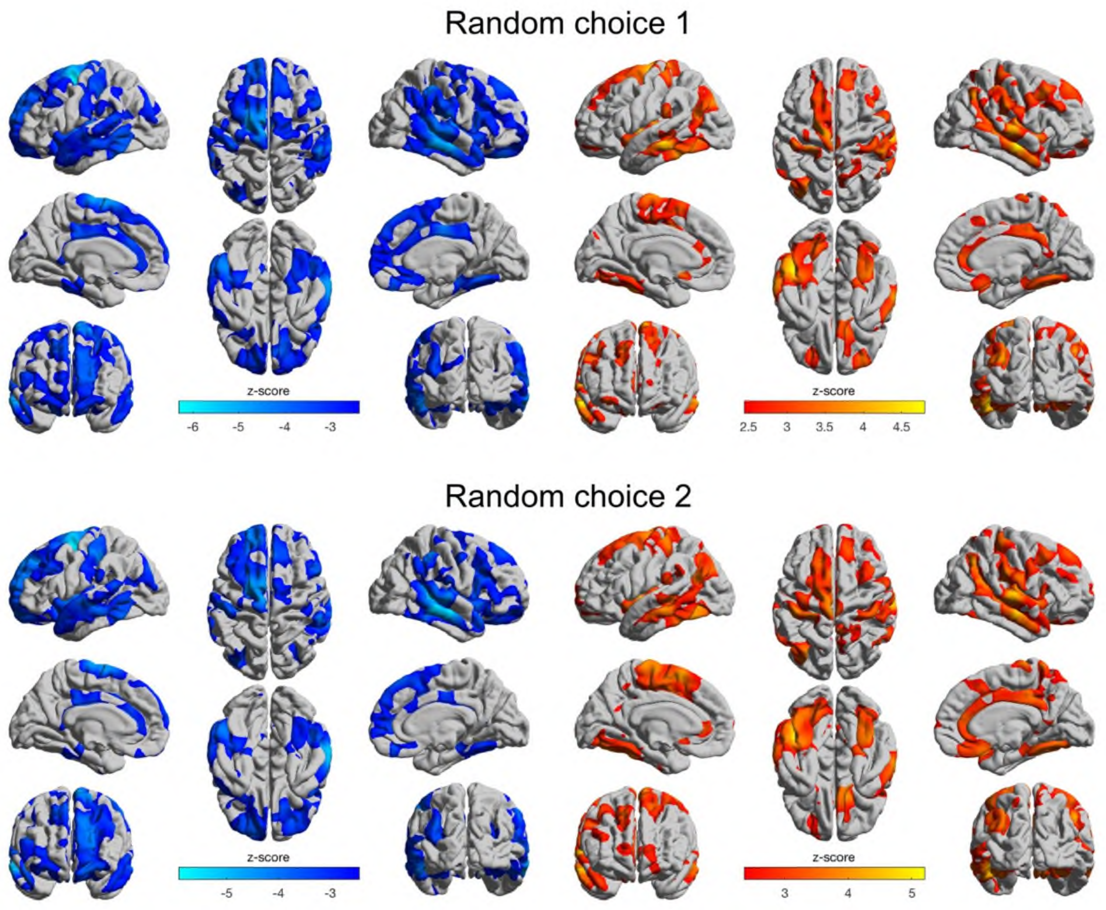

Supplementary Figure 2. The brain regions with their cortical volumes correlated with the family conflict score and parental monitoring score. a) The volumes of brain regions that were significantly correlated with the family conflict score (FDR p<0.05 corrected). The blue color indicates that lower cortical volumes were correlated with more severe family conflict scores. **b)** The volumes of brain regions that were significantly correlated with the parental monitoring score (FDR p<0.05 corrected). The red color indicates that higher cortical volumes were correlated with more positive parental monitoring.

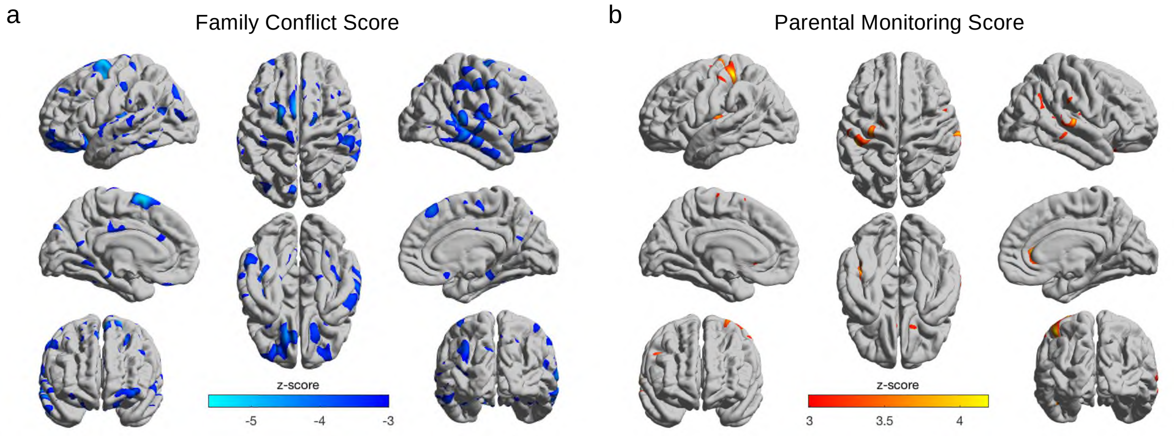

Supplementary Figure 3. a) A scatter plot of the vertex-wise z-statistics of the family conflict scores with cortical area and with cortical volume. Each point reflects the association between family conflict scores and the areas or volume of one vertex. **b)** A scatter plot of the vertex-wise z-statistics of the parental monitoring scores with cortical area and with cortical volume. **c)** A scatter plot of the vertex-wise mean cortical area and cortical volume.

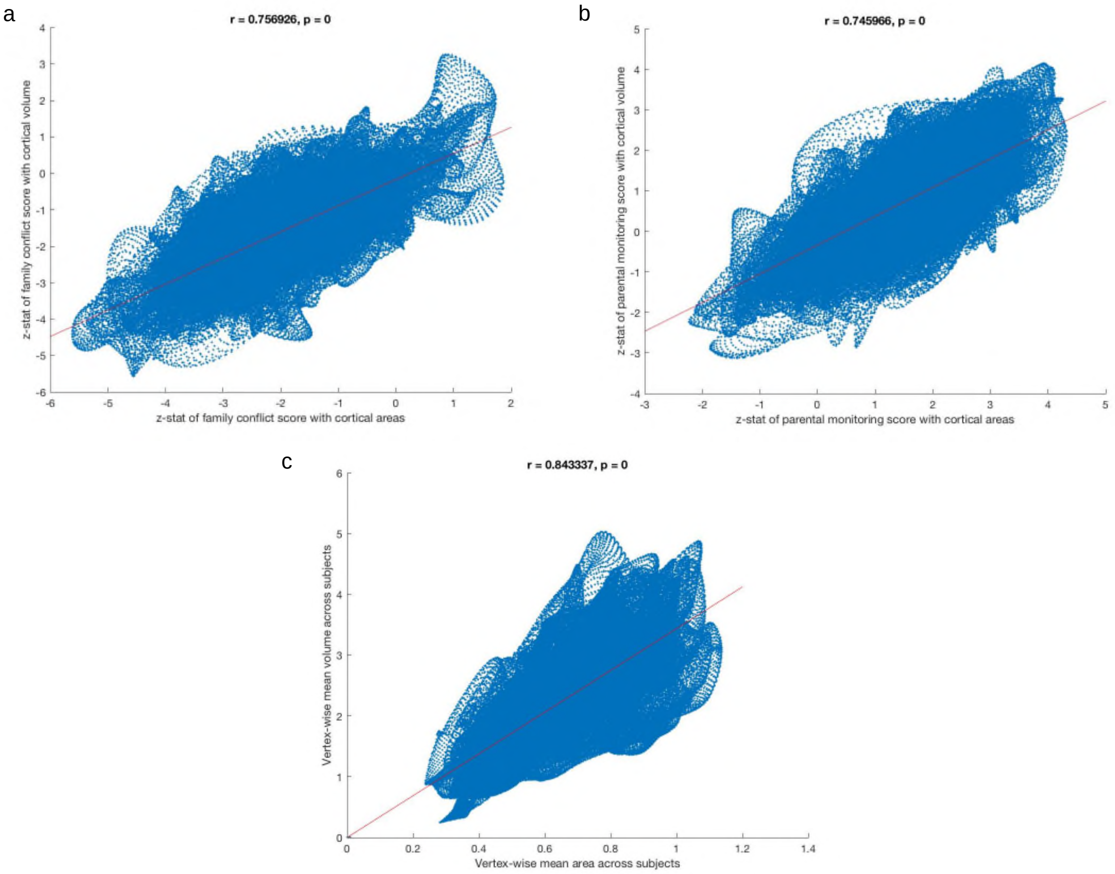

Supplementary Figure 4. A mediation analysis showed the cortical areas significantly mediate the association between the parental monitoring scores and behavioral problem scores. All statistical tests here are two-sided, and pass Bonferroni correction (p<0.05).

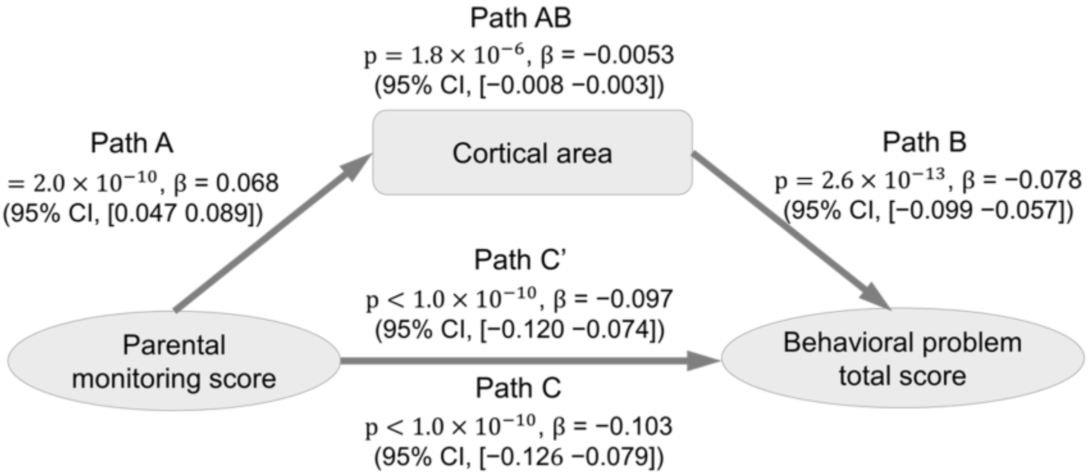

Supplementary Figure 5. a) A mediation analysis showed that the behavioral problem scores significantly mediate the association between the childrens' cortical area and the family conflict scores. b) A mediation analysis showed that the behavioral problems scores significantly mediate the association between the childrens' cortical area and the parental monitoring scores.

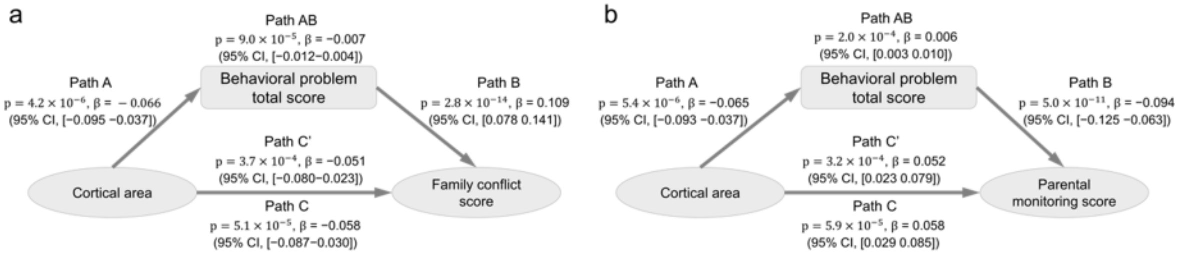

Supplement: Supplementary file 1 — Supplementary Information [file 41467_2021_23994_MOESM1_ESM.pdf]
