# Supplementary material for: Brain structure is linked to the association between family environment and behavioral problems in children in the ABCD study
Source: Nat Commun. 2021 Jun 18;12:3769. doi: 10.1038/s41467-021-23994-0 (PMC8213719; doi:10.1038/s41467-021-23994-0)
Supplement: Supplementary file 2 — Reporting Summary [file 41467_2021_23994_MOESM2_ESM.pdf]

nature research

Corresponding author(s): Wei Cheng

Last updated by author(s): Apr 26, 2021

Reporting Summary

Nature Research wishes to improve the reproducibility of the work that we publish. This form provides structure for consistency and transparency in reporting. For further information on Nature Research policies, see Authors & Referees and the Editorial Policy Checklist.

Statistics

For all statistical analyses, confirm that the following items are present in the figure legend, table legend, main text, or Methods section.

n/a	Confirmed	
☐	☒	The exact sample size (*n*) for each experimental group/condition, given as a discrete number and unit of measurement
☐	☒	A statement on whether measurements were taken from distinct samples or whether the same sample was measured repeatedly
☐	☒	The statistical test(s) used AND whether they are one- or two-sided<br>*Only common tests should be described solely by name; describe more complex techniques in the Methods section.*
☐	☒	A description of all covariates tested
☐	☒	A description of any assumptions or corrections, such as tests of normality and adjustment for multiple comparisons
☐	☒	A full description of the statistical parameters including central tendency (e.g. means) or other basic estimates (e.g. regression coefficient) AND variation (e.g. standard deviation) or associated estimates of uncertainty (e.g. confidence intervals)
☐	☒	For null hypothesis testing, the test statistic (e.g. *F*, *t*, *r*) with confidence intervals, effect sizes, degrees of freedom and *P* value noted<br>*Give P values as exact values whenever suitable.*
☒	☐	For Bayesian analysis, information on the choice of priors and Markov chain Monte Carlo settings
☒	☐	For hierarchical and complex designs, identification of the appropriate level for tests and full reporting of outcomes
☐	☒	Estimates of effect sizes (e.g. Cohen's *d*, Pearson's *r*), indicating how they were calculated

Our web collection on statistics for biologists contains articles on many of the points above.

Software and code

Policy information about availability of computer code

Data collection	No software was involved in data collection (data used is all directly available from ABCD, as described in detail in the paper).
Data analysis	Software used: Matlab 2019a, Freesurfer v6.0, Qoala-T v1.2, Mediation Toolbox v11/6/08 (https://github.com/canlab/MediationToolbox), MPlus v7.4. Custon code: https://github.com/weikanggong/ABCD_family_environment.

For manuscripts utilizing custom algorithms or software that are central to the research but not yet described in published literature, software must be made available to editors/reviewers. We strongly encourage code deposition in a community repository (e.g. GitHub). See the Nature Research guidelines for submitting code & software for further information.

Data

Policy information about availability of data

All manuscripts must include a data availability statement. This statement should provide the following information, where applicable:
- Accession codes, unique identifiers, or web links for publicly available datasets
- A list of figures that have associated raw data
- A description of any restrictions on data availability

Neuroimaging and behavioural data from ABCD dataset is obtained from https://nda.nih.gov/abcd with the approval of the ABCD consortium.

Field-specific reporting

Please select the one below that is the best fit for your research. If you are not sure, read the appropriate sections before making your selection.

☒ Life sciences ☐ Behavioural & social sciences ☐ Ecological, evolutionary & environmental sciences

Life sciences study design

All studies must disclose on these points even when the disclosure is negative.

Sample size	11,875 subjects in ABCD dataset. All currently available sample size were used in the study.
Data exclusions	For the 11,875 subjects, a total of 10,883 images were successfully preprocessed by Freesurfer. Quality control was then performed using software Qoala-T. 121 MRI images with scores below 40 were removed from the study, as recommended in the paper of the software. As there were correlated observations within families due to twins and siblings and at sites, we picked only one child in each family to eliminate this correlation effect in the subsequent statistical modelling, resulting in 9,117 children . After removing missing values in demographic and behaviour measures, data of 8,756 children were used in our analysis.
Replication	N/A. All available data are used to maximize statistical power of the analysis.
Randomization	N/A. This is an observational study.
Blinding	N/A. This is an observational study.

Reporting for specific materials, systems and methods

We require information from authors about some types of materials, experimental systems and methods used in many studies. Here, indicate whether each material, system or method listed is relevant to your study. If you are not sure if a list item applies to your research, read the appropriate section before selecting a response.

Materials & experimental systems

n/a	Involved in the study
☒	☐ Antibodies
☒	☐ Eukaryotic cell lines
☒	☐ Palaeontology
☒	☐ Animals and other organisms
☐	☒ Human research participants
☒	☐ Clinical data

Methods

n/a	Involved in the study
☒	☐ ChIP-seq
☒	☐ Flow cytometry
☐	☒ MRI-based neuroimaging

Human research participants

Policy information about studies involving human research participants

Population characteristics	A total of 11,875 participants aged between 9 to 11 years (52% males) was obtained from the ABCD study, which was a large longitudinal study that recruited children across 21 research sites across the USA. See Method section for further details. Population characteristics of the participants are listed in Table 1.
Recruitment	N/A (recruitment not part of this study)
Ethics oversight	Details can be found in the paper: Biomedical ethics and clinical oversight in multisite observationalneuroimaging studies with children and adolescents: The ABCD experience

Note that full information on the approval of the study protocol must also be provided in the manuscript.

Magnetic resonance imaging

Experimental design

Design type	Structural MRI
Design specifications	N/A
Behavioral performance measures	family conflict scores, parental monitoring scores, Youth NIH TB Summary Scores, Parent Child Behavior Checklist Scores

Acquisition

Imaging type(s)	Structural MRI
Field strength	3T
Sequence & imaging parameters	See ABCD datasets official websites for details.
Area of acquisition	Whole brain

Diffusion MRI ☐ Used ☒ Not used

Preprocessing

Preprocessing software	Freesurfer v6.0
Normalization	Freesurfer recon-all
Normalization template	fsaverage
Noise and artifact removal	N/A
Volume censoring	N/A

Statistical modeling & inference

Model type and settings	mass univariate, general linear model
Effect(s) tested	N/A

Specify type of analysis: ☒ Whole brain ☐ ROI-based ☐ Both

Statistic type for inference (See Eklund et al. 2016)	voxel-wise false discovery rate
Correction	FDR

Models & analysis

n/a	Involved in the study
☒	☐ Functional and/or effective connectivity
☒	☐ Graph analysis
☒	☐ Multivariate modeling or predictive analysis
